# Titanium Dioxide Nanoparticles: Grassian et al. Respond

**DOI:** 10.1289/ehp.10915R

**Published:** 2008-04

**Authors:** Vicki H. Grassian, Patrick T. O’Shaughnessy, Andrea Adamcakova-Dodd, John M. Pettibone, Peter S. Thorne

**Affiliations:** University of Iowa, Iowa City, Iowa, E-mail: vicki-grassian@uiowa.edu

Baveye and Laba have further analyzed the transmission electron micrograph (TEM) image shown in Figure 2A of our article (Grassian et al. (2007b) to quantitatively determine the extent of titanium dioxide nanoparticle clustering in the image by calculating the radial distribution function. The main point of doing this calculation was to demonstrate that TiO_2_ nanoparticle aggregates will not completely deaggregate even when subjected to harsh conditions.

We completely agree with the statement of Baveye and Laba that “aggregation may have toxicologic implications.” We disagree with their suggestion that “nanoparticle aggregation … so far has not been given serious consideration.” There is growing consensus that nanoparticle aggregation is an important factor in understanding the health implications of nanoparticles. This has been described by researchers working in the area of nanoparticle toxicity (Balbus et al. 2007; Powers et al. 2006), as well as by us. In addition to Grassian et al. (2007b), we refer to another study in which we further investigated TiO_2_ nanoparticle aggregation in inhalation and instillation studies (Grassian et al. 2007a). In that study we demonstrated that the size and nature of nanoparticle aggregates are important factors in evaluating their toxicity, and we suggested that the natural behavior of these particles and the manner in which people are exposed are critical factors in determining risk. If the nanoparticles do not deaggregate when inhaled, then the aggregation size and nature may be significant physicochemical properties in the toxicity of nanoparticles.

Moreover, combining extensive physicochemical characterization studies of nanoparticles with evaluation of their toxicity, as we have done (Grassian et al. (2007a, 2007b), will foster greater understanding of the environmental health impacts of nanotechnology.
